# Effectiveness of an integrated model of oral health-promoting schools in improving children's knowledge and the KAP of their parents, Iran

**DOI:** 10.1186/s12903-022-02644-x

**Published:** 2022-12-12

**Authors:** Bahareh Tahani, Imaneh Asgari, Shayan Golkar, Amir Ghorani, Nooshin Hasan Zadeh Tehrani, Fatemeh Arezoo Moghadam

**Affiliations:** 1 Department of Oral Public Health, Dental Research Center, Dental research Institute,School of Dentistry, Isfahan University of Medical Sciences, Isfahan, Iran; 2 Department of Oral Public Health, Dental Material Research Center, Dental Research Institute,School of Dentistry, Isfahan University of Medical Sciences, Isfahan, Iran; 3 Dental Students Research Commiittee, School of Dentistry, Isfahan University of Medical Sciences, Isfahan, Iran

**Keywords:** Knowledge, Attitude, Practice, Parents, School children, Dental health education

## Abstract

**Introduction:**

The aim of our study was to investigate the effect of an Oral Health Promoting School (OHPS) model on children's oral health in Iran.

**Methods:**

This interventional quasi-experimental study was conducted in the academic year 2019–20 among 354 primary school students and their parents. A questionnaire including 17 questions was distributed among children before and 5 months after the program (The ranges of possible scores = 0–17). Training workshops for the parents based on the theoretical domains framework were designed. Using educational sessions, pamphlets, tooth brushing dairies, assignments to do at home, educational videos and messages as reminders in social networks, parents were educated about dental caries, its risk factors and prevention principles. Best recommended oral health behaviors including tooth brushing and the use of fluoridated tooth paste were also educated. A questionnaire consisting of 18 knowledge (The ranges of possible scores = 0–18), 13 attitude and 10 practice questions were distributed among parents before and after the workshops. The data were fed into SPSS and analyzed by descriptive and analytic statistics such as T-test, ANOVA and Correlation Coefficients (α = 0.05).

**Results:**

The mean pre-test knowledge (7.8 ± 1.7) was increased significantly in three schools after program, *p* < 0.001. In the post-test, girls gained significantly higher scores (9.61 ± 1.98 vs. 9.06 ± 1.4, *p* = 0.025). Among 147 parents, the mean knowledge was raised from 12.3 ± 3.1 (5–18) to 15 ± 3.03 (6–18), *p* < 0.001. Knowledge score of the parents attending both sessions was higher. Practice of the parents regarding the use of fluoridated tooth-paste was significantly improved (*p* < 0.001). Also, their attitude toward the ability of children to take care of their teeth was improved (*p* = 0.029). Based on the self-report of parents, 71.4% (n = 47) of mothers and 45.6% (n = 67) of their children used to brush once or two times daily and there was a correlation between their behaviors (*p* < 0.001, Spearman Correlation Coefficient = 0.4).

**Conclusion:**

It seems that the education provided in OHPS had positive effects on increasing students' awareness and to some extent, the knowledge, attitude and practice of the parents.

## Introduction

Oral disorders such as dental caries and periodontal diseases are critical public health issues and the most common preventable chronic diseases of childhood [1]. Based on the results of the last National Oral Health Survey of Iran, conducted in 2016, the prevalence of dental caries in children aged 12 years was about 60%, the mean DFMT was 1.84, and the treatment need among 6 years old children was 76.28%, thus indicating that the prevention of dental caries is still a problem worthy of attention [2].

Untreated dental caries in children causes toothache, generalized pain and oral sepsis, leading to reduced food choices and interfering with the growth, as well as affecting the cognitive development of the child in the long term [3, 4]. In addition, dental caries have serious negative impacts on the social and psychological functioning in children, leading to school absences, inability to concentrate at school, reduced self-esteem, poor social relationships and impaired speech development [5]. It has been reported that poor oral health can lead to social exclusion and even hinder future employment, thereby reducing a child’s ability to succeed in life. On the other hand, improving child's oral health by reducing sugar consumption and regular tooth brushing with fluoride toothpaste might positively influence development, thus improving a child’s likelihood of engaging in learning at school [6].

Parents play a crucial role in maintaining and promoting their children's oral health. It is notable that young children’s health behavior is primarily steered by their parents, as children are unlikely to have the required capability and control over their own behaviors at home [7]. In addition, the way parents practice oral health for children is influenced by their beliefs and attitudes toward dental health, which is intrinsically shaped by their general cultural norms and value systems [8]. In this regard, a study on low-income African American preschool children revealed that children’s tooth-brushing frequency was significantly associated with their mothers’ knowledge of children’s oral hygiene. An increase in one unit of mothers’ knowledge score resulted in 13% increase in the tooth-brushing frequencies of 4–5 year children [9].

Children of parents with better oral health knowledge are more likely to have lower DMFT scores [10]. Besides this, parents’ attitudes toward diet and oral hygiene are identified as the risk indicators of dental caries in their children [11]. Parents act as a social model for their children; there is some evidence indicating the relationship between the oral health practices of the parents and their children [12, 13]. In a study done to investigate the pathways ranging from parental factors to oral health practices and status of children in Hong Kong, it was shown that children’s oral health status was directly affected by their mothers’ oral health behavior [14].

As oral health behaviors, beliefs and attitudes are shaped during childhood [15], schools are ideal environments for improving the oral health of children and adolescents. Schools can provide supportive measures to promote oral health, including policies and programs to increase schoolchildren's awareness and enhance school's safety to reduce facial and dental injuries, to improve student's nutrition patterns, to communicate with families, and to identify the children in need of appropriate dental treatment and then refer them to service centers [16]. The experience of running "Oral Health-Promoting Schools" in different parts of the world indicates oral health education programs in schools can serve as "cost-effective" interventions in terms of improving the attitudes, as well as oral health behaviors, especially in a short-term period [17, 18]. Accordingly, oral health education program continues to be developed and implemented in school settings; this necessitates the assessment and provision of cost-effectiveness evidence to stakeholders and decision makers. However, there is limited evidence regarding the implementation and assessment of such programs in Iranian schools. Therefore, the present study aimed to evaluate the implementation of an integrated model of OHPs in primary schools in regard to the changes in KAP (Knowledge, attitude and practice) of parents and the knowledge of schoolchildren. The effectiveness of the model in increasing the KAP of teachers has been published elsewhere [19].

## Materials and methods

This quasi-experimental before-after study was based on an agreement between the Vice Chancellery for Research at Isfahan University of Medical Sciences and the Deputy of Health and Research of the Department of Education, Isfahan Province, Iran, as an Action Research Plan with the Code of Ethics IR.MUI.RESEARCH.1397.1.012; it was conducted during 2018–2020 time period. Informed written consents were obtained from the parents of the recruited children for both educational and clinical interventions, separately.

### Participants

The minimum sample size for children was calculated based on the one sample paired design (before-after) formula [20]. By considering a standard deviation of 2.7 of changes in the knowledge of caries preventive behaviors in a similar study [21], the expected minimum effect size of 0.5, confidence interval of 95% and power of 80%, 231 samples were estimated to be needed for the present study. However, according to the design effect equivalent to 1.5 for cluster studies, the minimum sample size was calculated to be 346 samples. Besides this, by applying the same formula and considering the standard deviation of 2.6 in the knowledge of the parents based on a similar study [22] and the expected effect size of 0.7, at least 111 samples were needed for current study. By applying the design effect of 1.5 of cluster sampling, the sample size of the parents was calculated to be 166. (The design effect was calculated based on our previous study aimed to assess the knowledge of parents regarding fissure sealant and fluoride therapy [23] with the similar sampling design that the Intra Cluster Coefficient was 0.005 for about 567 participants using ANOVA).

Based on a multi-stage cluster sampling method, five primary schools of medium to low socioeconomic status were randomly selected from five educational regions of Isfahan city, Iran with a small size population (having up to 300 school children). All second-grade 7–8 years old students in the selected schools were recruited.

### Study design

**Fig. 1:**
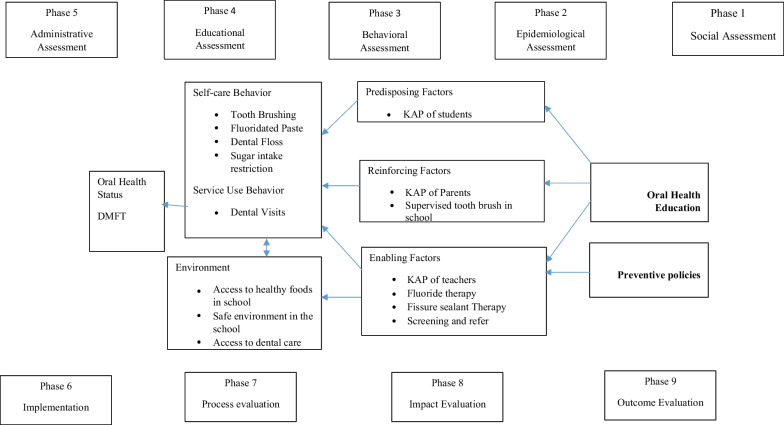
Conceptual map of the planning model based on the PRECEDE-PROCEED planning model

The protocol and details of the whole program as well as the interventions have been published previously [24]. Interventions were based on the multidimensional PRECEDE-PROCEED planning model [25]. Based on the previous studies on the school children’s oral health status and the factors affecting the oral health and effectiveness of interventions [17, 18], a conceptual map was designed based on the above model (Fig. 1) and the design of the “oral health-promoting schools” was adapted to the phases of this planning model; Phases 1–5 included the assessment of students' oral health status, parents' knowledge, attitudes and performance; assessment of teachers’ attitudes, performance and knowledge, as well as evaluation of schoolchildren’s oral health behaviors. Phase 6 included oral health education interventions for children, parents and teachers, professional screening, prevention and referral. Phase 7 included gathering the data related to the screened students’ numbers, the percentage of students receiving fissure sealant or fluoride, and the percentage of trained parents and teachers. Phase 8 consisted of assessing students’ improvement in knowledge and practice, teachers and mothers' oral health attitudes and behaviors, and brushing and flossing behaviors. Phase 9 included a cost analysis of implementing and monitoring the interventions that could be carried out using an economic method in future.

### Assessment phases

#### School children KAP (knowledge, Attitude, Practice)

A self-administered questionnaire was developed to elicit the children’s basic knowledge about oral health. The educational objectives of oral health promotion for this age group were set based on the literature review and some items were pooled for each objective. To ensure the face and content validity, the questions and educational objectives were given to 5 experts (3 oral public health and 2 dental pediatric professors). They were asked to rate the relevance of the questions based on a 3-point Likert scale (from 1: Completely relevant to 3: Not relevant). Questions were mostly about dental caries, risk factors, number of permanent teeth, approximate time of eruption, and duration and frequency of brushing. The reliability of the questionnaire was assessed by calculating the Guttman score (= 0.64) in a pilot study on 50 school children out of the project. The final questionnaire had 17 yes/no and open-ended questions and the final score could have been 0–17. In coordination with school principals, questionnaires were distributed among students before one of their formal classrooms by two dental students and gathered after 20 min. The dental students were available to further explain the questions in case there was difficulty understanding them.

To record the oral health status of the school children, clinical dental examination was done by some 5th year dental students (n = 30) using an electronic oral health software (*e-OHR*) [26] which was installed as a mobile application after instruction and training. Method of examining the teeth and recording caries indexes based on the WHO protocol [27] were taught to the dental students during two theoretical sessions in the year 3 as part of their “community oral health” and “caries diagnosis” courses, as well as a practical session in the year 4.

#### Parents KAP

A self-administered KAP questionnaire was developed and validated based on literature review [23, 28]. Knowledge questions were mostly multiple-choice questions, and some “yes", "no" and "don't know" questions. They were about the signs, symptoms and determinants of oral diseases and the oral health prevention methods and their effectiveness. Attitude questions were designed based on a five-point Likert scale (strongly agree to strongly disagree). The questions in the practice section were intended to assess the oral health behavior of parents and their children.

To ensure the face and content validity according to the method described in the school children section, the questionnaire was evaluated by 5 professors (2 at the Departments of Dental Pediatrics and 3 at the Oral Public Health Department). The reliability of the knowledge questions was assessed by a pilot study on 50 parents attending the pediatric clinic by using the split-half method (0.84). The reliability of the attitude questions in the pilot study was assessed by Cronbach's alpha (0.83). The final questionnaire consisted of 18 knowledge (The ranges of possible scores = 0–18), 13 attitude and 10 practice questions.

## Procedures (interventions phases)

### Schoolchildren training

Oral health issues were taught to the school children in several ways based on the health belief model [29].

**Fig. 2:**
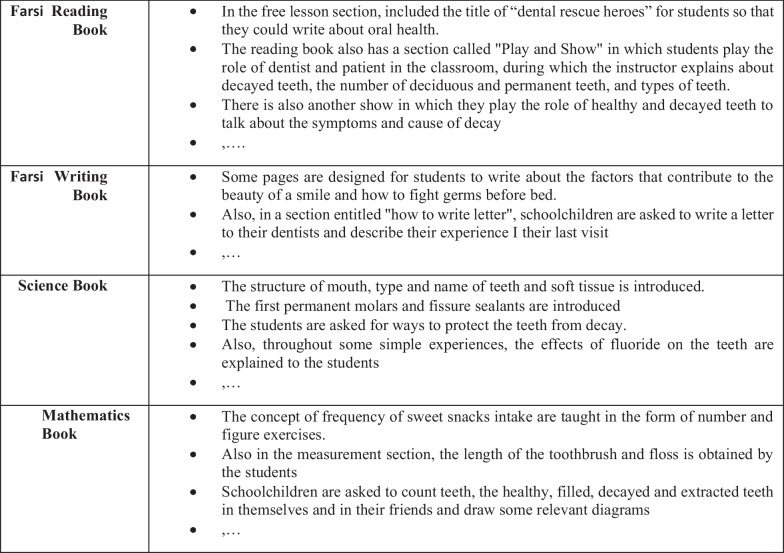
Examples of integrated oral health education contents into the primary schools' books

- *Face-to-face training* was theoretically and practically provided by the dental students in one session using colorful flip charts, big-size demonstration toothbrush and dental arches, disclosing tablets (Svenska Dentorama, Sweden), dental floss, disposable mirrors, soft toothbrush and fluoridated paste. Each student was given a toothbrush free of charge and after applying theoretical educations and using disclosing agents, they brushed their teeth under the supervision of dental students at schools’ yards. As part of their educational course, the dental students in semester 6 (year3) collaborated in this education, and each of the two dental students was responsible for 6 schoolchildren. The educational content was focused on the risk factors and consequences of dental diseases, preventive methods and diet recommendations. The whole training usually took 2 h.
- *Training through printed media*
  Worksheet 1: This worksheet included activities such as puzzles, cluttered words, tables, painting and crafting, all of which were related to the oral health topics. The worksheet content was based on the literature review and the educational tools in the website of ADA (American Dental Association). The face and content validity of this worksheet was confirmed based on the opinions of primary education experts in the provincial education council and oral public health experts. In art classes of schoolchildren, they were asked to fulfill one page of the worksheet weekly in 10 min.
  Worksheet 2: The basic oral health-related items were designed and integrated into different chapters of the elementary textbooks. The contents, and the selected books and chapters are shown in Fig. 2. This worksheet was also validated based on the opinions of the experts and the needed certification was got by the provincial education council. The protocol based on this worksheet was taught to the teachers in their workshops.
- *Motivational toothbrush and dental floss animation*
  This animation included teaching the acceptable oral health behaviors, including brushing (twice a day and two minutes each time using a fluoridated toothpaste) and flossing methods in the form of a kid song. The animation duration was exactly the amount of time (2 min) needed to brush.

### Providing preventive services to children

Children who were clinically examined and had healthy first or second molars with a moderate to high caries risk were selected for fissure sealant based on the last guidelines [30]. The list of students was sent to their parents for the inform consents (including some information about the number of teeth needed fissure sealant and the fact that the fourth-year dental students would provide this non-invasive care in the university's clinics). Parents were asked to take their children to the clinics themselves or by school principals. In the two educational clinics of the university, the dental students performed fissure sealants based on the last evidence for 1–4 teeth as needed for each child. All stages of the work, from tooth selection to the final stage of evaluation, were monitored by the supervising professors.

### Parent training

This included two 90-min workshop sessions, two printed media, and membership in a virtual Telegram channel. The training method included power point presentations, movies and discussion panels. The Theoretical Domain Frameworks model was then used to develop and design the parents’ educational content [31]. Based on the proposed 14 headings of this model, the methods to present the relevant content were selected according to the best available evidence and an expert panel including 3 oral public health and 2 health education experts. Accordingly, for the domain of “knowledge”, improving the parents' awareness of primary and permanent teeth development, and the determinants of dental diseases and the evidenced based recommendations [32] about tooth brushing and fluoride pastes (at least 1100 ppm and 1450 ppm for high risk children) through lecture was considered. Regarding the “skills” domain, through lectures, demonstrations and video presentations, parents were made familiar with oral hygiene instruments and the ways to clean the teeth, for the purpose of improving the self-confidence in the dental hygiene practice. To cover the domain of “social/professional role”, the responsibility of parents in regard to the oral health care of their children was outlined; to involve them more actively in this role, they were given tooth brush calendars and some information data sheets to observe their children's tooth brushing behavior, to check the plaque accumulation on teeth, and to evaluate the gingiva health status. To increase the self-confidence and self-capability of parents based on the domain of “beliefs about capabilities” through lectures, parents were given some examples and evidence regarding the effective role of parents in the prevention of dental diseases in children. According to the goals of the “beliefs about consequences” domain, through an interactive discussion and some videos and slides, parents were told about the consequences of oral diseases including abscess, pain, school absence, decreased learning abilities and the economic burden of dental diseases. For the domain of “reinforcement”, incentives like free oral health consultation, encouragement of children's participation and consideration of the remarks by the school administrates were taken into account. The domains of “intentions and goals” were covered through discussion panels and lectures. The short-term goals were set to improve the active supervision role of parents in regard to their children’s oral health behaviors and to sensitize them about the early symptoms of dental caries. The domain of “memory, attention and decision process” was followed by putting the content of each workshop in social media channel and the website of the dental school, giving two printed pamphlets and sending reminder messages to parents. According to the goals of the “environmental context and resources” domain, appropriate tooth brushes were provided to children; also, relevant posters and banners were given to introduce the program in the schools. Teachers and schoolchildren were also simultaneously engaged and educated. For the domains of “social influence” and “emotion”, we considered lectures to make parents aware of the positive role of oral health in social activities and communication, enhancement of the self-confidence, etc.

The videos and animations were designed by the dental students as part of their academic courses requirements based on the evidence-based recommendations [33, 34] or obtained from the available sources (after getting relevant permissions and citing the sources). The content and face validity of the prepared materials (power points, pamphlets and videos) were assessed by the expert panel (3 oral public health and 2 health education experts) using the relevant checklists published by the Ministry of Health in two areas including content and structure [35]. To evaluate the validity of the “content “of the materials, the coherence, relevancy attractiveness, acceptability, clearance and accuracy were checked using a Likert scale (1 = very low–5 = very high). Regarding structure, the design, text and quality of the production were checked. The total score of 100 for the content of each session was considered and the cut-off point of validity was determined as 70.

At the beginning of the workshop, the baseline KAP questionnaire was distributed. After obtaining the filled questionnaires, the educational content was provided; the first session was planned to provide an overview of oral and dental diseases, including caries and gingiva diseases and their determinants. Clinical methods for detecting dental caries, gingiva diseases, and dental plaque were also taught to parents through movies showing the use of disclosing tablets and introducing the early signs of gingivitis. At the end of the first session, parents were given a handbook and asked to assess their children's oral health behaviors within two weeks, including the frequency and duration of their brushing, and to fill out the toothbrush calendar. A colored chart was also provided to the parents to assess their children’s oral health status (dental caries and gum diseases) based on the instruction. They were given disclosing tablets and asked to check the status of their children’s dental plaque after one of their usual brushings.

The second training session was held two weeks later. In this session, they were introduced to the best practice of oral hygiene for children (tooth brushing and use of fluoride toothpaste) through lectures, slides and relevant videos. Parents were then asked to actively monitor their children’s brushing practice twice a day, record it in the brushing calendar and check their children’s dental plaque status, again with disclosing tablets after one month. The encouraging two-minute tooth brushing animation was then given to the parents through the social media channels.

For those who participated in the first session but were absent in the second one (group B), the content of the workshop, including the link and address of the website and a social media channel (Telegram), and the printed pamphlets and handbooks, was sent by the school administrators. The post-test KAP questionnaires were then distributed among parents one month after the workshop. The meetings were arranged in cooperation with the school board as part of the regular parent training sessions in the first week of the second semester. Training was provided by two senior dental students, a faculty member and a teacher in the school settings.

### Statistical Analysis and evaluation phase

The data was fed into SPSS (the post-test questionnaire was redistributed 5 months later) and questions related to the students’ practice were extracted and added based on their parents' statements. The Kolmogorov–Smirnov test was used to test the normal distribution of scores. The mean scores were compared by the paired t-test or its equivalent non-parametric test. The relationship between students' knowledge and health behaviors and their demographic characteristics was assessed using ANOVA and t-test or their equivalent non-parametric test. Pearson correlation was also used to assess the correlation between caries index and knowledge scores. For parents, the paired t-test was used to compare the pre- and post-intervention scores. ANOVA test was then conducted to compare the mean scores of knowledge based on the parents’ level of education and other factors. In all statistical analyses, the significance level was set at 0.05 and the non-parametric equivalent tests were used if the distribution of variables were not normal.

## Results

### Parents

From the population invited (about 350), 147 parents participated in the first workshop session. Demographic characteristics of the parents are demonstrated in Table 1. 143 (97.3%) were mothers with the mean age 36.1 ± 4.7(26–51). They had mostly two children and their education level was mostly diploma.

**Table 1:** Demographic characteristics of participated parents (N = 147)

	Frequency	Percentage
Education of father		
Illiterate	9	6.1
Under Diploma	51	34.7
Diploma	61	41.5
Academic education	26	17.7
Education of mother		
Illiterate	8	5.4
Under Diploma	35	23.8
Diploma	69	46.9
Academic education	35	23.8
Number of children		
1	33	23.9
2	81	58.7
3	15	10.9
4 or more	5	6.5
Participant from each school^*^		
A	35	23.8
M	22	15
K	27	18.4
N	33	22.4
S	30	20.4

*The full names of schools are hidden for ethical issues

### Practice

**Fig. 3:**
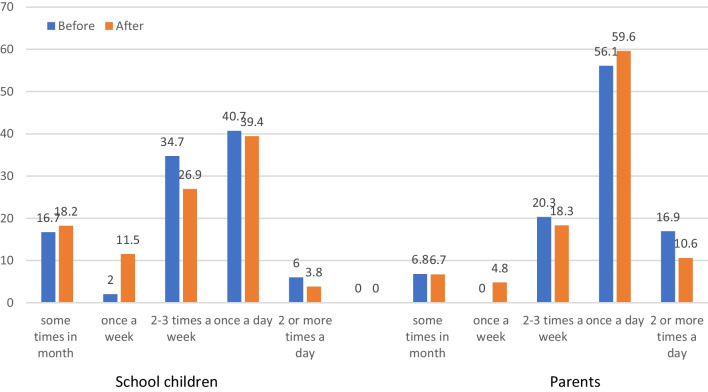
Distribution of tooth brushing behavior among parents and their children reported by parents before and after the program implementation

Self-reported tooth brushing behavior of parents and their children before and after the program is presented in Fig. 3. At the baseline, it was found that most of the children (about 40%) and parents (about60%) brushed once daily. Frequency of the parents’ answers to the practice questions also revealed that 24 percent of them reported the pattern of sugar between-meals consumption as twice or more a day. Meanwhile, 36% of them reported that they devoted their attention to the concentration of fluoride when they were buying the appropriate toothpaste for the family. Also, 23% of them reported supervising the efficiency of their child tooth brushing regularly; further, about 16% had almost never assessed the dental and gingival health of their kids. The frequency for the parents using fluoridated toothpaste for their children was 50.7 too.

After the interventions, the frequency of the parents using fluoridated toothpaste for their children was increased to 65% (*p* = 0.008). In addition, their attention to the concentration of fluoride was raised to 68.6% following the workshops (*p* < 0.001). Changes in other practice schemes was not, however, significant. By considering once or twice a day as the cut-off point for the regular tooth brushing [36], 71.4% (n = 47) of mothers and 45.6% (n = 67) of schoolchildren had this practice. There was a significant correlation between their behaviors (*p* < 0.001, Spearman Correlation Coefficient = 0.4). According to the Phi test, 83% of mothers with irregular tooth brushing reported this behavior for their children as well. On the other hand, 57.1% of mothers with regular tooth brushing reported the same behavior in their children.

### Knowledge

The mean of the parents’ knowledge was significantly raised from 12.3 ± 3.1 (5–18) to 15 ± 3.03 (6–18), Wilcoxon, *p* < 0.001. In all 5 schools, the knowledge mean scores was increased significantly (Table 2); however, the difference between them was not significant. In addition, parents who participated actively in both workshops (group A) gained a significantly higher score, as compared to those who participated in the first session and received the educational content of the second session (group2); in group A, the mean knowledge was increased significantly from 12.8 ± 3.2 to 15.2 ± 3.1 (*p* < 0.001), while in group B, the mean knowledge was raised from 13.6 ± 2.5 to 14.4 ± 2.8 (*p* = 0.018).

**Table 2:** The baseline and post-intervention knowledge among participated school children (n = 354) and their parents (n = 147) in Isfahan, Iran

Schools^*^	School children			Parents			SES of the school
	Pre-test	Post-test	*P* value^**^	Pre-test	Post-test	*P* value^**^	
A	8.1 ± 7.8	9 ± 3.2	0.13	10.6 ± 3.4	14.5 ± 3.1	< 0.001	Low
M	7.6 ± 1.6	10.5 ± 1.9	< 0.001	13.3 ± 3.4	16.8 ± 2.2	< 0.001	Moderate
S	7.7 ± 1.6	9.8 ± 1.7	< 0.001	12.8 ± 2.4	14.8 ± 3.7	0.004	Low to moderate
K	6.9 ± 1.5	9.1 ± 0.9	0.001	12.6 ± 3.2	15 ± 3.1	0.004	Moderate
N	8.4 ± 1.6	8.9 ± 1.2	0.06	12.7 ± 2.7	14.4 ± 2.2	0.007	Low

*The full names of schools are hidden for ethical issues

**Comparison of pre and post-test knowledge scores

The percentage of correct answers to knowledge questions is presented in Table 3. At the baseline, the least frequency (25%, n = 37) of correct answers to knowledge questions was related to the recommended and optimal frequency of daily tooth brushing. In contrast, the highest frequency of the correct answers (84%, n = 123) was gained for the questions related to the role of sugary snacks in dental caries and the positive role of regular tooth brushing in the prevention of gum diseases (85%, n = 124). After the workshops, the percentage of correct answers was increased, especially in regard to the best advice for brushing and role of dental plaque in dental caries.

**Table 3:** percentage of pre and post-test correct answers regarding the knowledge questions among participated parents (N = 147)

Knowledge themes	Percentage of correct answers	
	Pre-test	Post-test
1. Best advice for brushing	25.2	54.4
2. Best advice for toothbrush type	71.4	90.5
3. Dental Caries symptoms	79.6	85
4. Bleeding during brushing	61.6	73
5. Risk of drinking soft drinks	74.8	89.8
6. Effects of early primary teeth extraction	66.7	76.9
7. Effects of primary teeth infection	48.3	69.4
8. Risk of permanent caries among those with primary teeth caries	54.2	79.6
9. Role of genetic in dental caries	51.7	79.6
10. Role of dental plaque in caries	59.9	87.8
11. Role of dental plaque in gingivitis	59.9	79.6
12. Role of sugary between-meal snacks in dental caries	70.1	83.7
13. Effectiveness of toothpaste	70.1	92.5
14. Role of fluoride in caries prevention	79.6	98
15. Role of mouth rinse in caries prevention	63.3	86.4
16. Importance of sugar consumption restriction in caries prevention	84.4	95.9
17. Importance of regular tooth brushing in gingivitis prevention	85.7	94.6
18. Linkage between oral health and general health	81	85.7

ANOVA test also showed a significant difference between the baseline knowledge of mothers and their level of education (*p* < 0.001). Those who were illiterate (7.5 ± 2.8) or under Diploma (11.1 ± 2.9) got lower knowledge scores, as compared to those who had academic education (13.2 ± 2.9). However, there was no correlation between mothers’ education and the mean difference of knowledge scores following the interventions. A positive correlation between the mean difference of knowledge and age of parents (*p* = 0.023, Spearman Correlation Coefficient = 0.7) and the number of children in the family (*p* = 0.042, Spearman Correlation Coefficient = 0.2) was obtained. In addition, there was a positive correlation between the mean knowledge of parents and their tooth brushing behavior (acceptable/unacceptable, *p* < 0.001, Coefficient = 0.4). Parents with regular habits had higher knowledge scores (12.3 ± 3.1 vs. 10.7 ± 3.3, *p* = 0.008).

### Attitude

**Fig. 4:**
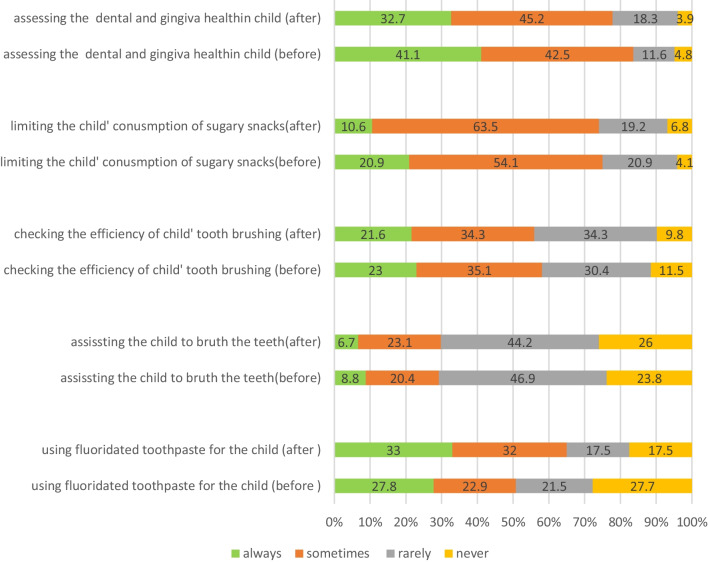
Attitude of parents regarding oral health before and after the implemented workshops (N = 147)

According to the responses recorded for the attitude section (Fig. 4), at the baseline, 89% (n = 130) of the parents found the pleasant sense of mouth after brushing a highly encouraging factor. Most of them considered the importance of oral diseases as other general diseases and 15% (n = 22), partially or completely, agreed that edentulousness in old people was a natural accident and thus, inevitable. Also, 15% (n = 22), partially or completely, agreed that primary teeth were not very important as they would be exfoliated, regular tooth brush would result in teeth and gum destruction, and the supervision of parents would have negative and inhibitory effects on their children. Although about 80% (n = 117) of parents believed schools could be good places for the implementation of oral hygiene educations, 45% (n = 66) were concerned about the time and other facility limitations in schools. On the other hand, one third of the parents believed a 3 year old kid should take the responsibility of her/his dental self-care.

After workshops, attitude scores underwent significant changes, especially in terms of the perception of the parents regarding the ability of 3-year-old kids to brush their teeth without help (*p* = 0.029). Also, the number of parents holding a negative attitude about the active supervision of parents (15.7% to 6.1%, *p* = 0.023), and the gum and teeth destructive effects of tooth brushing was decreased (*p* = 0.012). Parents' preference regarding other statements did not change significantly.

### Schoolchildren

354 children (girls 38.7%, n = 137) from 5 primary schools participated in the study and their baseline information were gathered. In the post test days, 246 (response rate = 70%) were available. Some of the students were absent since it was near the final exams.

At the baseline, the mean knowledge scores was 7.8 ± 1.7 and increased significantly to 9.4 ± 1.8 (*p* < 0.001) after the interventions (Table 2). There was no difference in the pre-test scores based on gender (7.66 ± 1.74 vs. 7.96 ± 1.8); However, in the post-test, girls gained significantly higher scores (9.61 ± 1.98 vs. 9.06 ± 1.4, *p* = 0.025). As the pre test scores were different, the percentage of changes in post test scores, as compared with the pre-test scores, was calculated, showing that in two schools the changes were significantly higher (M = 50%, K = 42%, *p* < 0.001). The least changes were recorded, in two schools which had the lowest social and economic status (N = 11.5%, A = 21%), based on the reports of the Province Education Council.

The dental carries status of children, based on the electronic records, showed that the total DMFT and dmft means were 1.2 ± 1.7 and 5.9 ± 3.5, respectively, and no significant difference was reported between schools. Regarding the preventive care, fissure sealant therapy was provided for 209 children, which included 577 teeth in the dental clinics of the dental school in two different geographic regions of the city by dental students free of charge. The percentage of attending students, among those who were invited to receive fissure sealant, showed that the need for fissure sealant therapy ranged from 79 to 92% in our schools. In one of the schools (K) whose records were kept more accurately, from 40 students in a class, 36 needed FS (90%) and so, they were invited; on days of clinical care, 25 students attended with their parents (69.4%). Finally, they received 68 fissure sealants for their teeth (1–4 teeth/student).

## Discussion

In the current study, we implemented a type of oral health promoting school model using the potentials of dental school to improve the KAP of parents and schoolchildren as a means to improve the oral health. We used the TDF model to design and implement the interventions for parents. It has been reported that the domains that have gained the most attention are usually knowledge and beliefs, while other important domains such as emotions, intentions and social roles have been neglected [37]. We tried to propose special and feasible interventions to cover these domains as well. Despite the proved crucial role of parents in shaping a regular tooth brushing behavior in their children, to guide parents in promoting this practice, it is also important to go beyond simple knowledge transmission to support their intentions to supervise children's toothbrushing [38].

In our study, the knowledge scores were increased significantly; also, the attitude scores changed according to the beliefs of the parents regarding the ability of kids to perform their oral self-care and their negative attitude toward the supervision of parents. Further, it seemed that the implemented model was successful in improving the knowledge and attitudes of parents in regard to the importance of fluoride concentration in tooth paste and their practice in becoming more actively involved in the tooth brushing behavior of their children. While other practice patterns did not change dramatically in our study, Bin Peng et al. [39], in their oral health promoting school model, could enhance the practice pattern of parents with regard to sugar consumption, regular tooth brushing, active supervision of children’s tooth brushing and the possibility of assisting them. Their study, however, was implemented in three years, during 30 sessions; thus, it benefited from reinforcement more than our study did.

Hashemian et al. [40], in an oral health promoting model, also sent some cell phone messages in their intervention group, beside providing pamphlet contents, reporting its effectiveness in increasing the knowledge of parents. We also used this method by sharing the relevant educational contents through our virtual group and sending short reminder messages to parents that could partly justify their knowledge improvement. Naidu et al. [41] and Brown et al. [42] also indicated the effectiveness of even face-to-face and in-depth discussion sessions in schools in increasing the knowledge and reshaping the attitude of parents.

In our study, the increase of knowledge scores was not correlated to the educational level of parents, but there was a correlation between the educational level of mothers, their baseline knowledge scores and the oral health status of their children; so, the more education, the more the knowledge scores and the more the chance of filling in the primary teeth. These findings were, thus, in agreement with those of Chen et al. [43], in which parents with a good educational background were found to have more favorable oral health knowledge and their children had better oral hygiene behaviors. This achievement could be explained by the relationship between oral health literacy (OHL) and educational level of mothers. It is shown that mothers’ OHL is significantly linked to their oral hygiene practices and their children’s preventive behavior (brushing) and filling treatment [44]. In our study, mothers with a higher educational level reported a better oral hygiene behavior, both in themselves, and in their children and themselves, thus indicating the mediating role of OHL, although we did not assess this indicator.

Furthermore, regarding the schoolchildren, the results indicated the effective role of our interventions in increasing the knowledge of them. This finding was in agreement with many other studies implemented in school settings, especially those benefiting from multi-method interventions such as playing, drawing and face-to-face education [45]. In addition, in schools with a better socio-economic status, the mean difference of knowledge was increased more significantly. This finding has been shown in other previous studies as well [46], indicating the lower awareness, oral health practice and lower dental visits at schools in low SES regions. This also necessitates prioritizing the implementation of oral health programs in these regions using targeted population approaches.

Gender difference was another finding in our study; girls gained higher knowledge scores, which was in agreement with other similar studies; this, thus, showed that girls had better scores in their oral health knowledge and attitudes. This could be partly explained by the higher positive attitude and attention of girls to their appearance, encouraging them to attain more knowledge about their oral health and care more [47, 48].

Regarding the behavior of tooth brushing, 4% and 40% of children in our study reported brushing twice a day and once a day, respectively; so, this seems inadequate when compared to the frequency of 77% [39] or about 60% for the rate of twice a day, as reported in other studies [49]. According to the proved role of regular tooth brushing, especially twice a day, in preventing dental caries [50], this behavior should be reinforced more among children. Supervised-tooth brushing programs are recognized as effective interventions in school settings that should be established in schools having access to the proposed infrastructure [51]. Regarding the schools considered in our study, at the time when this program was started, such infrastructure including enough space and appropriate bathroom sinks, trained teachers and needed permissions were not available. However, this intervention is considered for the upcoming versions of our program and we are now conducting feasibility studies by getting support from the main stakeholders in the Province Educational Council.

## Conclusion

Overall, the effectiveness of the proposed integrated model of OHPS was assessed in 5 schools as pilots. Although the results were not promising in terms of behavior change and might have limitations due to quasi-experimental designs and other constraints such as considering short-term goals (it was not possible for us to assess the incidence of new caries due to COVID 19 lock-downs after one year), inability to calibrate dental students as examiners thoroughly (due to the course time limitations) and inability to increase sample size and recruiting control matched group, it offered some characteristics making its establishment more reasonable. It is an interacting model meeting the academic requirements of dental schools and implementing preventive programs in primary schools. Considering the number of dental schools in the country and current dental students (about 2000), this model can be beneficial to both schoolchildren in deprived schools and also, dental students. In addition, this model could simultaneously involve parents, teachers, children and Educational Council administrators.

Although we considered such intermediate short-term goals as knowledge, attitude and behavior, in a recent systematic review, to assess the effectiveness of school-based oral health promotion programs, it was shown that comprehensive programs implementing educational programs for children and parents, considering oral examination, provision of fluoride toothpaste, and preventive and curative treatments, could also significantly lower DMFS increment mean score and sulcus bleeding scores, thus contributing to the changes towards the good practices of oral care, as compared to the control group in the long term [52].

## Data Availability

The datasets used and/or analyzed during the current study are available from the corresponding author on reasonable request. Due to the fact that the study is before-after and the name of students and parents are included, for confidentiality reasons, the data are not publicly available.
